# Assessing Consumer Perceptions of Foods from Insect-Fed Animals in the Kingdom of Saudi Arabia: A Cross-Sectional Study

**DOI:** 10.3390/foods15213891

**Published:** 2026-10-30

**Authors:** Thamer Alhasyani, Saleh Altuwaijri, Hamad Alhumaidan, Mansour Almutairi, Hassan Barakat

**Affiliations:** 1 Department of Pathology and Laboratory Diagnosis, College of Veterinary Medicine, Qassim University, P.O. Box 6622, Buraydah 51452, Saudi Arabia; 461115654@qu.edu.sa (T.A.); stoiejry@qu.edu.sa (S.A.); 2 Saudi Food and Drug Authority, Northern Ring Rd, Hittin, Riyadh 13513, Saudi Arabia; hmhumidan@sfda.gov.sa (H.A.); mh.mutairi@sfda.gov.sa (M.A.); 3 Department of Food Science and Human Nutrition, College of Agriculture and Food, Qassim University, Buraydah 51452, Saudi Arabia

**Keywords:** insect-protein, consumer acceptance, food safety, halal concerns, sustainability, food supply

## Abstract

This study examined consumer perceptions, acceptance, and perceived barriers to products derived from animals fed insect-based protein in Saudi Arabia, a topic relevant to sustainable protein production and future feed regulations. A cross-sectional questionnaire survey was conducted using convenience sampling of a structured Arabic instrument that assessed sociodemographic characteristics, prior awareness, post-information attitudes, acceptance, and concerns. The survey measured perceptions of safety, regulatory trust, environmental benefits, and economic considerations and assessed predefined barriers, including insufficient knowledge, religious concerns, disgust, food-safety concerns, allergy risk, and possible sensory changes. Results showed relatively high awareness of insect protein and its nutritional and environmental values. After brief exposure to information, respondents generally expressed favorable attitudes, with many indicating a willingness to consume, purchase, or try products from insect-fed animals. Acceptance increased when products were perceived as safe, officially approved, environmentally beneficial, and halal-certified, with halal certification emerging as the condition receiving the highest level of agreement on purchase intention. Price also influenced willingness to buy, with stronger acceptance at prices equal to or below conventional products and only moderate willingness to pay a small premium. However, higher price increases reduced acceptance. Overall, respondents in this convenience sample reported generally favorable attitudes toward insect-based feed following brief informational exposure; however, these findings should not be generalized to all Saudi consumers. The study recommends public education, transparent regulation, and clear labeling to support market introduction and consumer confidence in products from animals fed insect-based protein.

## 1. Introduction

The demand for animal-derived protein continues to rise worldwide, placing significant pressure on agricultural production systems and natural resources [1]. According to FAO [2], agricultural production must increase by nearly 70% by 2050 to feed a global population expected to reach 9.7 billion people, while simultaneously reducing environmental damage and addressing climate change. Although the livestock sector provides billions of people worldwide with essential proteins and nutrients, maintaining this supply sustainably remains a major global challenge. This increasing demand continues to place pressure on the livestock sector to expand production sustainably while minimizing environmental impacts. Moreover, over the period 2005–2050, global demand for meat and dairy products is expected to increase by 70–80%, while demand for crop protein is expected to increase by 100–120% [3]. Most people prefer to obtain protein from animal sources because it is an essential nutrient that supports growth, tissue repair, and normal metabolic functions in the human body [4].

Other protein sources, such as soybean meal and fishmeal, are among the most commonly used in animal feed. However, both are associated with negative environmental effects. For instance, growing soybeans contributes to the destruction of biodiversity hotspots such as the Amazon rainforest. Similarly, overfishing threatens marine ecosystems and makes fishmeal difficult to obtain and expensive. In addition, climate change may affect the sustainability of these conventional protein sources through changes in rainfall patterns, extreme weather events, and crop productivity [5]. Therefore, many studies on sustainable alternative protein sources for animal feed have been conducted, and interest has increased in recent years.

Insects are a good source of protein for animal feed. Many insect species possess complete amino acid profiles that are as good as or better than those of regular protein sources. Mealworms can have as much as 52% crude protein, while black soldier fly larvae can have as much as 42%. Insects are a rich source of amino acids that animals need, especially lysine and methionine, which are often lacking in their diets [6]. Using insect-based protein feed offers several ecological benefits compared with conventional protein sources. Insects require less land, water, and energy for production [7]. They contribute to circular economy systems by converting organic waste into protein and reducing waste disposal costs. In addition, several life-cycle assessment studies have reported that insect production systems require less water and land and generate lower greenhouse gas emissions than conventional production systems [8]. Several insect species have shown strong potential as alternative protein sources for animal feed. When grown on organic waste, black soldier fly (*Hermetia illucens*) larvae provide poultry and fish with high-quality protein. Yellow mealworms (*Tenebrio molitor*) are rich in protein and can be fed to many different animals. In addition, crickets and grasshoppers are consumed in many cultures and are rich in essential nutrients, making them suitable for both human and animal nutrition [9]. Insect-based protein feed can be divided into three categories: (1) live insects, (2) processed animal proteins (PAPs), and (3) fats, chitin, and other derived products [10].

Despite the nutritional and environmental advantages of insect-based protein, its successful adoption depends largely on consumer acceptance. Insect protein may be more acceptable in mainstream food systems through “indirect entomophagy”—eating products from insects-fed animals. Given the environmental and nutritional benefits, people may be more likely to eat insect-fed meat, eggs, and dairy than to consume insects directly, according to preliminary studies [11]. Therefore, several studies have been published regarding consumer acceptance of insect-based protein feed and related products. In Western countries, very few consumers are familiar with eating insects, and many people reject the idea because they find it disgusting [12]. Hungarian consumers showed higher acceptance among males, highly educated individuals, and respondents aged 30–39 years [13]. Interestingly, a study conducted in Italy demonstrated that increasing consumer awareness regarding the benefits of insect-based protein feed can positively influence consumer acceptance and purchasing intention [14]. These findings indicate the need for greater efforts to improve environmental awareness, health perceptions, and trust in food safety authorities. In addition, consumer attitudes may also be influenced by knowledge of the ecological, nutritional, and production benefits of insect-based protein feed. Conducting this research and collecting consumer feedback in the Kingdom of Saudi Arabia may help inform future regulations on the use of insect-based protein feed. While several regions, such as the European Union, have established regulatory frameworks for the production and use of insect-based protein feed, regulatory policies in Saudi Arabia remain limited or unclear [10].

Beyond safety and sustainability considerations, the acceptability of insect-protein animal feed in Saudi Arabia is shaped by Islamic dietary law (fiqh al-at’imah), which governs the permissibility (halal status) of animal-based foods. The classification of insects as halal or haram is not uniform across Islamic jurisprudence and remains a matter of scholarly debate among the four Sunni schools of thought. The Maliki school is generally permissive toward the consumption of non-harmful insects, while the Hanafi school is the most restrictive, classifying most land insects as khabith (repulsive) and therefore impermissible; the Shafi’i and Hanbali schools occupy an intermediate position, permitting select species under specific conditions [15]. Notably, all four Sunni schools agree that locusts are halal, based on hadith evidence in Sunan Ibn Majah (Hadith 3314), which explicitly permits fish and locusts as two exceptions to the general prohibition on consuming dead animals without ritual slaughter (Sunan Ibn Majah 3314, [16]). Because Saudi Arabia’s religious and legal framework is predominantly informed by Hanbali jurisprudence, this doctrinal context is directly relevant to how consumers may evaluate the acceptability of animal products derived from insect-fed livestock, even when the insects themselves are not directly consumed. Prior research in other cultural and religious contexts has shown that religion is among the primary factors shaping consumer rejection of insect-containing foods, alongside disgust and food-safety concerns [17,18]. Studies conducted in other Muslim-majority or insect-relevant settings, such as Lebanon and India, have similarly emphasized that religious and cultural frameworks meaningfully shape acceptance of entomophagy-related products [19]. This religious dimension motivates the present study’s inclusion of halal-related items and underscores the need to interpret findings on consumer acceptance within this doctrinal framework.

This study addresses informational gaps by providing consumer approval ratings across various product categories and cultural contexts in Saudi Arabia. The study provides valuable insights for industry stakeholders, policymakers, and researchers regarding consumer attitudes, perceptions, purchasing intentions, and willingness to support sustainable protein production systems.

## 2. Materials and Methods

### 2.1. Study Design and Setting

A cross-sectional, questionnaire-based study was conducted to evaluate consumer acceptance of animal-derived food products fed insect-based protein in Saudi Arabia. The study was designed to characterize consumer knowledge, perceptions, acceptance, and perceived barriers at a single point in time, which is consistent with the purpose of survey-based observational research.

### 2.2. Study Population and Eligibility

The target population comprised adult individuals residing in Saudi Arabia. The target population comprised adult Saudi residents aged 18 years and above. A non-probability convenience sampling approach was used, with the questionnaire link shared electronically and respondents encouraged to disseminate it further within their networks. Inclusion criteria were Saudi residency, age ≥ 18 years, and voluntary informed consent; incomplete responses were excluded from the final dataset. Eligible participants self-selected into the study after reading the introductory information describing the study purpose, expected duration, and confidentiality assurances. Participants were invited to complete the survey voluntarily after reading the introductory information provided at the beginning of the questionnaire.

### 2.3. Questionnaire Development and Structure

The questionnaire was developed after reviewing previous studies on consumer acceptance of insect-based food and feed. Items were selected and adapted to cover prior awareness, perceived safety, regulatory trust, environmental benefits, halal considerations, purchase intentions, price sensitivity, and consumption barriers. Face and content validity were assessed by 4 experts in food science, consumer research, survey design, and Islamic dietary regulations. The experts evaluated each item for relevance, clarity, cultural appropriateness, and coverage of the study objectives. Their comments were incorporated into the revised questionnaire. The questionnaire was pilot-tested with 20 adults meeting the eligibility criteria. Participants assessed the clarity, wording, response options, questionnaire length, and technical functionality. Based on their feedback, the questionnaire was modified. Pilot participants were not included in the final analysis. The attitudinal items assessed distinct dimensions and conditions of acceptance rather than forming a single psychometric scale. Cronbach’s alpha was also used to assess the internal consistency of the acceptance items, yielding a coefficient of 0.87.

Data was collected using a structured Arabic-language questionnaire developed specifically for the present study. The instrument was organized into five sections. The first section recorded sociodemographic characteristics, including sex, age group, education level, geographic region within Saudi Arabia, and monthly income category. The second section assessed prior knowledge of insect protein use in animal feed, sources of prior information, awareness of nutritional and environmental benefits, pre-existing reservations regarding consumption of products from insect-fed animals, awareness that insects may constitute a natural component of some animal diets, and awareness of the halal status of such products in Saudi Arabia.

It should be noted that this item captured respondents’ general awareness of halal status and one conditional-purchase item tied to halal certification; the instrument did not assess respondents’ specific knowledge of the jurisprudential (fiqh) rules governing insects as a feed source, nor their level of religious observance or madhab affiliation. These items were therefore designed as proxies for perceived regulatory-religious trust rather than direct measures of religiosity or doctrinal knowledge, a scope decision we revisit in the Discussion and Limitations.

The third section consisted of a brief standardized information phase delivered via a short text presentation. According to the questionnaire, this text presentation introduced participants to the environmental, economic, and nutritional benefits of using insect protein as an alternative feed ingredient, including reduced greenhouse gas emissions, conservation of natural resources, and potential benefits for animal and consumer health. The fourth section measured post-exposure attitudes toward consuming products from animals fed insect-based protein using a 5-point Likert scale ranging from 1 (“strongly disagree”) to 5 (“strongly agree”). The fifth section assessed the primary concern or reason that could limit respondents’ acceptance of food products obtained from animals fed insect-based protein. Respondents selected one response from a predefined list comprising no concerns, insufficient knowledge or need for additional information, religious considerations, feelings of disgust, food-safety concerns, potential allergic reactions, possible changes in sensory characteristics, and other factors. No open-ended question was included in the questionnaire. The full distributed questionnaire in its original Arabic language was provided as Supplementary Material.

### 2.4. Information Exposure

Before the attitude assessment, participants were shown a short text presentation outlining the rationale for using insect-based protein in animal nutrition. This standardized exposure was intended to ensure that responses to the acceptance items were provided after a minimal, uniform level of topic-specific information had been presented to all respondents. Incorporating a brief educational component before assessing attitudes is an established approach in survey research when the topic may be unfamiliar to part of the study population. Therefore, the subsequent attitude responses were interpreted as post-information responses rather than spontaneous or unprompted consumer attitudes.

### 2.5. Study Variables and Outcome Measures

The primary outcome of the study was consumer acceptance of food products obtained from animals fed insect-based protein. This construct was operationalized through questionnaire items assessing willingness to purchase such products if available on the market, to consume meat or eggs from animals fed insect-based protein, and to try such products if a free trial were offered. Additional acceptance-related items evaluated whether environmental benefits, circular-economy considerations, perceived healthfulness, perceived safety, official regulatory approval, replacement of genetically modified soybean feed, and contributions to sustainability and food security in Saudi Arabia would increase product acceptance.

The questionnaire also assessed economic acceptance through items examining willingness to purchase products when priced similarly to conventional products, willingness to pay a slightly higher price if health or environmental benefits were perceived, and unwillingness to purchase if the price exceeded that of conventional products by more than 10%. Secondary variables included prior awareness of insect protein in animal feeding, reported sources of knowledge, pre-existing reservations, and awareness of halal considerations. Perceived barriers were assessed using a structured, closed-ended, single-response item. Respondents were asked to select the primary concern or reason that would make them unwilling or hesitant to consume products obtained from animals fed insect-based protein. The predefined response options were insufficient knowledge or need for additional information, religious considerations, feelings of disgust, food-safety concerns, potential allergic reactions, concerns about changes in sensory characteristics, no concerns, and other factors.

### 2.6. Response Format

Attitudinal items were measured using a 5-point Likert-type response format, with higher scores indicating greater agreement and, by extension, greater acceptance. Likert scaling is widely used in consumer and health-related survey research to assess attitudes, beliefs, and behavioral intentions quantitatively. The barrier item used a single-response categorical format in which respondents selected the one option that best represented their primary concern. This format was used to identify the most prominent barrier reported by each respondent.

### 2.7. Data Collection Procedure

The questionnaire was administered electronically using Google Forms between March and June 2026. The survey link was disseminated through multiple channels, including social media platforms, university and professional networks, and community/consumer groups across Saudi Arabia’s five administrative regions, to maximize reach across diverse demographic segments. Respondents accessed the survey voluntarily via the shared link and were free to circulate it further within their own networks. The questionnaire introduction informed participants that completion would require approximately 15–20 min and stated that participation was voluntary. The introductory statement also emphasized that all collected information would remain confidential and be used solely for scientific research and publication. After reading this information and agreeing to proceed, respondents completed the demographic and prior-knowledge sections, viewed the short text presentation, and then answered the post-exposure attitude and barrier items in the sequence defined by the questionnaire.

The questionnaire was developed and administered electronically in Arabic to ensure clarity and accessibility for the target population in Saudi Arabia. For publication, the Arabic questionnaire was translated into English by bilingual members of the research team. The English version was subsequently reviewed against the original Arabic questionnaire to ensure linguistic accuracy, conceptual equivalence, and consistency of meaning. Any discrepancies were resolved through discussion among the researchers before the final English responses were included in the manuscript.

### 2.8. Ethical Considerations

The study was conducted in accordance with the Declaration of Helsinki. It was approved by the Sub-committee of Health and Bioethics Research Ethics, Vice Rectorate for Graduate Studies and Scientific Research, Qassim University, Saudi Arabia (approval No. 26-13-2; 25 March 2026). Informed consent was obtained electronically from all participants before survey completion.

### 2.9. Statistical Analysis

Data were analyzed using descriptive statistics. Categorical variables are presented as frequencies and percentages, while responses to the Likert-scale items are presented according to the distribution of responses across the five response categories. The structured barrier item is reported as the frequency and percentage of respondents selecting each predefined response option. Associations between the defined acceptance outcome and respondent characteristics were assessed using Pearson’s chi-square tests of independence. Statistical significance was defined as *p* < 0.055. No qualitative or thematic analysis was performed because the questionnaire did not include an open-ended question as described by Steel et al. [20].

## 3. Results

### 3.1. Participant Characteristics

Table 1 summarizes reservations and acceptance by respondents’ sociodemographic characteristics. Before receiving information, 43.36% reported no reservations, 33.33% reported reservations, and 23.31% were unsure. After information exposure, 58.76% agreed that they would consume meat or eggs from insect-fed animals, while general acceptance reached 64.41%. Acceptance was broadly similar by sex and education but varied descriptively by age, income, and region. Meat-or-egg acceptance was highest among respondents aged 18–29 years (75.90%), those earning below 5000 SAR (64.21%), and residents of the Southern region (63.75%). General acceptance was highest in the Southern region (70.00%) and lowest in the Central region (55.88%). General acceptance also exceeded acceptance of the specific meat-or-egg item by 5.65 percentage points. Overall, positive responses exceeded negative responses across most demographic groups, indicating generally favorable attitudes in this sample. However, these unadjusted descriptive differences do not establish statistically significant associations or independent predictors; appropriate cross-tabulation tests and multivariable analysis are required to support stronger interpretations.

### 3.2. Prior Awareness and Knowledge of Insect-Based Feed

Data in Table 2 indicated that most respondents (*n* = 567, 80.08%) reported prior awareness of the use of insect protein as a protein source in animal feed, whereas 141 respondents (19.92%) had not previously heard of this concept. Among those reporting prior awareness, the most common source of knowledge was academic studies (*n* = 175, 24.72%), followed by news or articles (*n* = 152, 21.47%), social media (*n* = 147, 20.76%), other sources (*n* = 126, 17.80%), friends or family (*n* = 95, 13.42%), and occupation-related exposure (*n* = 13, 1.84%). Knowledge of the nutritional and environmental value of insect protein was also relatively high. A total of 545 respondents (76.98%) reported being aware that feeding animals insect protein may provide nutritional benefits, such as high protein content, as well as environmental benefits. Before information exposure, 43.36% of participants reported no reservations about consuming products from animals fed insect-based protein, while 33.33% had reservations, and 23.31% were unsure. In addition, 86.86% recognized that insect protein is a natural component of the diet of some animals. Both findings were statistically significant (*p* = 0.048 and *p* < 0.001, respectively).

### 3.3. Attitudes Toward Safety, Official Approval, and Environmental Benefits

Hence, perceived safety was highly accepted; the data in Table 3 illustrate attitudes toward safety, official approval, and environmental benefits. However, when asked whether knowing that insect protein is safe and would not harm health would increase the likelihood of consuming such products, 460 respondents agreed or strongly agreed (64.97%), including 321 who agreed (45.34%) and 139 who strongly agreed (19.63%). By contrast, 80 respondents (11.30%) disagreed or strongly disagreed, whereas 168 (23.73%) were neutral.

Confidence in regulatory approval was even stronger. A total of 485 respondents (68.50%) agreed or strongly agreed that they would feel reassured if these products were safe and approved by official authorities, while only 75 respondents (10.59%) disagreed or strongly disagreed; 148 respondents (20.90%) selected a neutral response. Environmental framing also positively influenced responses. A total of 442 respondents (62.43%) agreed or strongly agreed that environmental benefits would encourage them to consume products from animals fed insect-based protein, compared with 106 respondents (14.98%) who disagreed or strongly disagreed; 160 respondents (22.60%) remained neutral.

The sustainability narrative received strong support. Overall, 504 respondents (71.19%) agreed or strongly agreed that the use of insect protein in animal feed contributes to the circular economy and enhances sustainability and food security in Saudi Arabia, whereas 76 respondents (10.74%) disagreed or strongly disagreed, and 128 (18.08%) were neutral. The halal-related item received the highest agreement among the assessed conditional-purchase items; however, this does not establish halal certification as an independent predictor of purchase intention. A total of 505 respondents (71.32%) agreed or strongly agreed that they would buy animal products from animals fed insect-based protein if such products were considered halal under Islamic law in Saudi Arabia. In comparison, only 91 respondents (12.85%) disagreed. Given the doctrinal contestation surrounding insect permissibility discussed in the Introduction, this result suggests that halal status functions not merely as one attitude item among several, but likely as an underlying moderating factor shaping responses to related items on safety, regulatory trust, and willingness to pay—an interpretation we develop further in the Discussion.

Respondents also showed favorable attitudes toward replacing genetically modified protein sources with insect-based protein in animal feed. In total, 434 respondents (61.30%) agreed or strongly agreed that such substitution would increase their willingness to consume meat from these animals, whereas 98 (13.84%) disagreed or strongly disagreed, and 183 (25.85%) were neutral. Direct acceptance of consuming meat or eggs from animals fed insect-based protein was generally positive. A total of 416 respondents (58.76%) agreed or strongly agreed that they would accept consuming such products, including 247 (34.89%) who agreed and 169 (23.87%) who strongly agreed. In contrast, 124 respondents (17.51%) disagreed or strongly disagreed, and 168 (23.73%) were neutral. General acceptance was likewise favorable. When asked whether they were generally comfortable with consuming animal products from animals fed insect-based protein, 456 respondents (64.41%) agreed or strongly agreed, whereas 104 respondents (14.69%) disagreed or strongly disagreed, and 148 (20.90%) reported neutral views.

### 3.4. Purchase Intention and Market Availability

The data in Table 4 relate to purchase intention and market availability. Purchase intention under routine market conditions was also positive. A total of 445 respondents (62.86%) agreed or strongly agreed that they would buy animal products from animals fed insect-based protein if these products were available in the market. Conversely, 111 respondents (15.67%) disagreed or strongly disagreed, while 152 respondents (21.47%) were neutral. A free-trial strategy was also well received. Overall, 445 respondents (62.59%) agreed or strongly agreed that they would try these products if given a free trial; 106 (14.97%) disagreed or strongly disagreed; and 157 (22.18%) were neutral.

Price clearly influenced consumer willingness to purchase. When asked whether they would buy animal products from animals fed insect-based protein if these products were cheaper than traditional products, 455 respondents (64.26%) agreed or strongly agreed, whereas 111 respondents (15.68%) disagreed or strongly disagreed, and 142 respondents (20.06%) were neutral. A moderate willingness to pay a premium was also observed. A total of 380 respondents (53.67%) agreed or strongly agreed that they would pay a slightly higher price if the products were beneficial to human health; 155 (21.89%) disagreed or strongly disagreed; and 173 (24.44%) were neutral. Similarly, 396 respondents (55.94%) agreed or strongly agreed that they would pay a slightly higher price if the products contributed to environmental conservation and emission reduction, compared with 142 respondents (20.06%) who disagreed or strongly disagreed and 170 respondents (24.01%) who remained neutral.

However, resistance became more apparent at higher price increments. When asked whether they would not buy these products if the price exceeded that of traditional products by more than 10%, 298 respondents (41.95%) agreed or strongly agreed, 188 (26.55%) disagreed or strongly disagreed, and 223 (31.50%) selected a neutral response. In general, 456 respondents (64.41%) agreed or strongly agreed that they were okay with consuming animal products from animals fed insect-based protein, whereas 104 (14.69%) disagreed or strongly disagreed, and 148 (20.90%) were neutral; this distribution was statistically significant (χ^2^ = 33.374, *p* < 0.001).

### 3.5. Reported Barriers to Consumption

The distribution of respondents’ primary concerns is presented in Table 5. Of the 708 respondents, 328 (46.33%) selected “no concerns.” The most frequently reported specific barrier was insufficient knowledge or the need for additional information (*n* = 121, 17.09%), followed by religious considerations (*n* = 99, 13.98%), feelings of disgust (*n* = 81, 11.44%), and food-safety concerns (*n* = 66, 9.32%). Potential allergic reactions (*n* = 6, 0.85%), concerns about changes in sensory characteristics (*n* = 5, 0.71%), and other factors (*n* = 2, 0.28%) were reported less frequently. These results represent responses to a predefined, closed-ended item and should not be interpreted as findings from qualitative or thematic analysis.

## 4. Discussion

The findings indicate that foods derived from animals fed insect-based protein may be socially acceptable in Saudi Arabia, but acceptance depends on consumer trust, cultural compatibility, and market conditions. In sustainable protein transitions, public confidence in food safety, ethical legitimacy, and affordability is as important as nutritional and environmental performance. Recent Saudi studies likewise show that willingness to accept insect-based proteins is shaped by sociocultural barriers, food neophobia, and perceptions of safety and religious permissibility [21,22,23].

The halal-related item received the highest agreement among the assessed conditional-purchase items; however, this does not establish halal certification as an independent predictor of purchase intention. Because insect permissibility is doctrinally contested across Islamic legal schools, and Saudi Arabia follows predominantly Hanbali jurisprudence, this aggregate figure likely masks a spectrum of religious interpretations rather than a uniform stance. Stricter respondents may weigh halal compliance more heavily than less strict or non-Muslim respondents. As the instrument did not stratify by religious observance, madhab affiliation, or fiqh knowledge, this pooled estimate should be read as an average across an unmeasured range of religious strictness, not a homogeneous response. Future studies should incorporate validated scales of religiosity and Islamic dietary law knowledge to disaggregate this effect.

The respondents’ demographic profile was skewed toward middle-aged, highly educated women from across Saudi Arabia, which is relevant because acceptance of novel proteins often varies by age, education, and socioeconomic status. Prior research shows that knowledge, exposure to sustainability messages, and familiarity with alternative proteins are linked to greater openness. At the same time, younger and more educated respondents are often more receptive to innovation [24,25]. The significant differences across age, education, region, and income indicate that attitudes toward insect-fed animals are not uniform and should not be addressed with a single communication strategy. Instead, public campaigns should be tailored to specific demographic groups, with emphasis on trust, clarity, and cultural reassurance for less familiar segments of the population [24,25,26].

The relatively high prior awareness of insect protein in animal feed is notable, given that insect-based feed remains a relatively new concept in many food systems. Academic studies, media, and social platforms appear to be the primary channels of information, suggesting that both formal and informal sources shape consumer familiarity. Previous work also shows that prior knowledge and exposure to information are important drivers of acceptance of novel foods, especially when the environmental and nutritional rationale is clearly communicated [27,28,29]. A substantial share of respondents already recognized the nutritional and environmental value of insect protein and understood that insects naturally form part of some animals’ diets. This perception may reduce the sense of unnaturalness that often limits acceptance of emerging food technologies. Consistent with earlier research, indirect entomophagy is generally more acceptable than direct insect consumption because respondents perceive the insect component as part of animal feed rather than human food. However, the persistence of reservations among a sizeable minority indicates that awareness alone is not sufficient for full acceptance, underscoring the need for more targeted education and trust-building strategies [25,27,28,30].

Table 3 shows that higher agreement was observed for items concerning mainly perceived safety, official approval, environmental benefit, and halal status. Safety is especially important because perceived health risk is a common barrier to the adoption of novel foods, and respondents tend to accept innovations more readily when scientific evaluation and health assurance are clear [28,31]. Trust in regulation was equally central, since certification and official endorsement often serve as proxies for credibility when respondents cannot directly evaluate the production process. Environmental framing also improved acceptance, consistent with evidence that sustainability messages can strengthen openness to alternative proteins, especially when they are combined with safety reassurance rather than presented alone [31,32,33]. However, because these attitudes were assessed after respondents had viewed positively framed information about the potential benefits of insect-based feed, the responses may not fully reflect spontaneous attitudes. The strong effect of halal status is particularly relevant in Saudi Arabia, where permissibility is a core criterion of food legitimacy and trust. Finally, the favorable response to replacing genetically modified protein sources suggests that insect-based feed may be viewed as a more natural or acceptable alternative to conventional inputs, supporting its positioning as a practical, socially acceptable, sustainable protein option [23,25,34,35].

The results indicate that willingness to buy and try insect-fed products is higher when the products are available on the market or offered as a free trial, which fits evidence that information, exposure, and trialability can reduce hesitation toward novel foods. Free-trial approaches may therefore help bridge the gap between expressed acceptance and actual purchase behavior [24,36,37,38]. Price also strongly shapes acceptance, with greater willingness when insect-fed products are priced at or below conventional alternatives and weaker demand when premiums become too large. This is consistent with sustainable food research showing that consumer enthusiasm often declines as perceived financial sacrifice increases, even when environmental benefits are acknowledged [38,39]. Taken together, the findings suggest that consumer acceptance is highest when safety, sustainability, affordability, and religious acceptability are communicated together rather than separately. Accordingly, producers and marketers should use integrated messaging that combines environmental benefits, official approval, halal certification, and competitive pricing to move respondents from interest to routine purchase [24,32,34].

The structured barrier assessment showed that 46.33% of respondents reported no concerns about consuming products obtained from animals fed insect-based protein. Among the predefined barriers, insufficient knowledge or the need for additional information was the most frequently selected, followed by religious considerations, feelings of disgust, and food-safety concerns. These findings are consistent with previous research identifying unfamiliarity, religious acceptability, emotional aversion, and perceived health risks as relevant influences on the acceptance of insect-related foods [27,32,40,41]. The results suggest that consumer communication should provide accessible information about product safety, feed production, regulatory oversight, and halal compliance. However, because respondents selected only one predefined response, the findings identify the distribution of primary concerns but do not capture the possible coexistence or complexity of multiple barriers. Sensory concerns are also valid, since appearance and texture are often stronger barriers than taste, and future sensory studies should test product quality under market-relevant conditions [42,43,44]. These findings suggest that insect-based feed could be a practical route for alternative protein innovation, especially where direct insect consumption is culturally sensitive. They also show that consumer education works best when it is backed by trusted institutions, clear regulation, and culturally appropriate messaging. For industry and policymakers, gradual introduction, strong labeling, safety assurance, halal certification, and a clear national regulatory framework are essential for successful adoption [45].

The study is limited by its cross-sectional design, reliance on self-reported data, and context-specific sample, which may limit generalizability and inflate positive responses. Future research should use longitudinal, experimental, and mixed-methods approaches to examine how attitudes evolve and whether they translate into real purchasing behavior, while also testing different message frames, product types, price levels, labeling formats, halal cues, and sensory acceptance under market conditions. However, these findings underscore that religious compliance, interpreted in the context of Islamic dietary law, is central to consumer acceptance of insect-protein animal products in Saudi Arabia, and that future research should further disaggregate this effect by level of religious observance.

The assessment of consumption barriers was based on a structured, closed-ended item in which each respondent selected one primary concern. The questionnaire did not include an open-ended question and therefore could not capture detailed explanations, additional concerns, or the possible coexistence of multiple barriers. No qualitative or thematic analysis was performed. However, several limitations should be considered when interpreting these findings. First, the cross-sectional, convenience-sampled design limits causal inference and generalizability, as the electronic, self-selected recruitment approach likely over-represents digitally engaged, more educated respondents. Moreover, 89.41% of respondents had university or postgraduate education, which may have contributed to the high level of prior awareness and may limit the applicability of the findings to less-educated groups. Moreover, 89.41% of respondents had university or postgraduate education, which may have contributed to the high level of prior awareness and may limit the applicability of the findings to less-educated groups. Second, the study did not measure respondents’ religious observance, madhab affiliation, or specific knowledge of Islamic jurisprudential rulings on insect consumption; consequently, associations between halal-related items and acceptance should be interpreted as an average effect across an unmeasured and likely heterogeneous distribution of religious strictness and may not generalize uniformly across more versus less observant sub-populations or to non-Muslim residents. Third, the attitudes were self-reported and measured after a brief informational exposure; therefore, they may not represent spontaneous opinions or predict actual purchasing behavior. Fourth, although the instrument demonstrated face and content validity, it had not been previously applied in a Saudi halal-food context and may benefit from further psychometric validation, including a dedicated religiosity/halal-knowledge subscale. Future studies should include clearly documented open-ended questions or qualitative interviews and apply transparent coding and reliability procedures to explore these issues in greater depth. Also, additional research should incorporate validated scales for religiosity and Islamic dietary law knowledge to disaggregate responses by the strictness of religious observance, which the current dataset cannot support.

## 5. Conclusions

This study provides evidence on the attitudes of respondents in this Saudi convenience sample toward foods from insect-fed animals, indicating meaningful market potential when safety, official approval, environmental benefits, and halal compliance are assured, with halal certification emerging as the condition receiving the highest level of agreement on purchase intention. These findings warrant caution: the convenience-sampled, electronically recruited design likely over-represents digitally engaged, more educated respondents, and because religious observance, madhab affiliation, and jurisprudential knowledge were not measured, halal-related associations should be read as an average across an unmeasured, heterogeneous spectrum of religious strictness rather than a uniform response, while self-reported, single-timepoint attitudes may not fully translate into real purchasing behavior. These findings suggest potential consumer acceptance within this sample, but nationally representative studies are required before conclusions can be extended to the wider Saudi population. Future research should employ longitudinal, mixed-methods designs incorporating validated scales of religiosity and knowledge of Islamic dietary law to disaggregate responses by observance and to test message framing, pricing, and halal-labeling cues under real-world market conditions.

## Figures and Tables

**Table 1:** Associations between respondent characteristics and general acceptance of products from insect-fed animals.

Characteristic	Category	Prior Reservations No	Unsure	Yes	Meat/Egg Acceptance Agree	Neutral	Disagree	General Acceptance Agree	Neutral	Disagree
Sex	Male	138	79	110	219	92	70	237	79	65
	Female	169	86	126	197	76	54	219	69	39
Age (years)	18–29	94	47	54	148	24	23	131	40	24
	30–44	137	82	115	180	87	67	208	81	45
	45–59	67	32	57	88	34	34	101	24	31
	≥60	9	4	10	0	23	0	16	3	4
Education	Secondary or less	35	12	28	46	17	12	48	13	14
	University	214	111	163	284	115	89	311	107	70
	Postgraduate	58	42	45	86	36	23	97	28	20
Monthly income (SAR)	No income	39	14	22	48	20	7	52	14	9
	<5000	50	21	24	61	19	15	67	16	12
	5000–10,000	108	46	65	134	48	37	145	47	27
	>10,000	50	34	59	80	33	30	85	29	29
	Prefer not to disclose	60	50	66	93	48	35	107	42	27
Region	Southern	35	18	27	51	18	11	56	17	7
	Eastern	54	35	43	76	33	23	87	27	18
	Northern	51	24	43	65	33	20	82	20	16
	Western	81	33	60	108	39	27	117	38	19
	Central	86	55	63	116	45	43	114	46	44
Total	All respondents	307	165	236	416	168	124	456	148	104

Note: Values are frequencies. For acceptance outcomes, “Agree” combines “strongly agree” and “agree,” while “Disagree” combines “disagree” and “strongly disagree.” For prior reservations, “No” indicates no reservations before information exposure.

**Table 2:** Prior awareness and knowledge of respondents (*n* = 708).

Questions	Items	Numbers (*n*)	Ratio %	Chi^2^ Value	*p*-Value
Have you ever heard of using insect protein as a protein source in animal feed?	Yes	567	80.08	36.000	0.001
	No	141	19.92		
What is the source of knowledge regarding the use of insects as a protein source in Animal feed?	Academic studies	175	24.72	22.273	0.001
	News or articles	152	21.47		
	Social media	147	20.76		
	Friends or family	95	13.42		
	The job	13	1.84		
	Other	126	17.80		
Did you know that feeding animals insect protein achieves nutritional benefits (such as high protein content) and also environmental benefits?	Yes	545	76.98	29.160	0.001
	No	163	23.02		
Before learning any information, do you have any reservations about consuming animal products from animals fed insect-based protein?	Yes	236	33.33	6.610	0.048
	No	307	43.36		
	Not sure	165	23.31		
Did you know that insect protein is a natural part of the diet of some animals (e.g., worms for birds, larvae for fish, or certain types of insects for poultry)?	Yes	615	86.86	54.760	0.001
	No	93	13.14		

**Table 3:** Attitudes toward safety, regulation, and sustainability (*n* = 708).

Questions	Items	Numbers (*n*)	Ratio %	Chi^2^ Value	*p*-Value
Knowing that using insect protein as an alternative protein source in animal feed is safe and will not harm my health increases the likelihood that I will consume these products	Strongly agree	139	19.63	53.300	0.001
	agree	321	45.34		
	Neutral	168	23.73		
	Disagree	50	7.06		
	Strongly disagree	30	4.24		
I feel reassured knowing that these products are safe and approved by official authorities	Strongly agree	242	34.18	41.300	0.001
	agree	243	34.32		
	Neutral	148	20.90		
	Disagree	55	7.77		
	Strongly disagree	20	2.82		
The environmental benefits will encourage me to consume products from animals fed insect-based protein	Strongly agree	179	25.28	33.000	0.001
	agree	263	37.15		
	Neutral	160	22.60		
	Disagree	77	10.88		
	Strongly disagree	29	4.10		
I believe that using insect protein as animal feed contributes to the circular economy and enhances sustainability and food security in Saudi Arabia	Strongly agree	223	031.5	48.158	0.001
	agree	281	39.69		
	Neutral	128	18.08		
	Disagree	47	6.64		
	Strongly disagree	29	4.10		
I will buy animal products from animals fed insect-based protein if they are halal according to Islamic law in Saudi Arabia	Strongly agree	268	37.85	43.700	0.001
	agree	237	33.47		
	Neutral	112	15.82		
	Disagree	51	7.20		
	Strongly disagree	40	5.65		
I believe that replacing protein sources with insect-based protein in animal feed increases my acceptance of consuming the meat of these animals	Strongly agree	197	26.84	30.000	0.001
	agree	237	33.47		
	Neutral	183	25.85		
	Disagree	62	8.76		
	Strongly disagree	36	5.08		
I would accept consuming meat or eggs from animals fed insect-based protein.	Strongly agree	169	23.87	25.583	0.001
	agree	247	34.89		
	Neutral	168	23.73		
	Disagree	83	11.72		
	Strongly disagree	41	5.79		

**Table 4:** Attitudes toward purchase intention, price, market availability, safety, regulation, and sustainability (*n* = 708).

Questions	Items	Numbers (*n*)	Ratio %	Chi^2^ Value	*p*-Value
I will buy animal products from animals fed insect-based protein if they are available on the market.	Strongly agree	165	23.31	36.707	0.001
	agree	280	39.55		
	Neutral	152	21.47		
	Disagree	67	9.46		
	Strongly disagree	44	6.21		
I will try these products if I am allowed to try them for free.	Strongly agree	165	23.31	34.889	0.001
	agree	280	39.28		
	Neutral	157	22.18		
	Disagree	62	8.76		
	Strongly disagree	44	6.21		
I would buy animal products from animals fed insect-based protein if they were cheaper than traditional products.	Strongly agree	189	26.69	32.812	0.001
	agree	266	37.57		
	Neutral	142	20.06		
	Disagree	64	9.04		
	Strongly disagree	47	6.64		
I am willing to pay a slightly higher price if these animals’ products from animals fed insect-based protein are beneficial to human health.	Strongly agree	161	22.74	17.111	0.002
	agree	219	30.93		
	Neutral	173	24.44		
	Disagree	95	13.42		
	Strongly disagree	60	8.47		
I am willing to pay a slightly higher price if these animal products, from animals fed insect-based protein, contribute to environmental conservation and emissions reduction	Strongly agree	166	23.45	19.030	0.001
	agree	230	32.49		
	Neutral	170	24.01		
	Disagree	87	12.29		
	Strongly disagree	55	7.77		
I will not buy these products if the price is more than 10% higher than the price of traditional products.	Strongly agree	119	16.67	16.300	0.003
	agree	179	25.28		
	Neutral	223	31.50		
	Disagree	129	18.22		
	Strongly disagree	59	8.33		
In general, I am okay with consuming animal products from animals fed insect-based protein.	Strongly agree	198	27.97	33.374	0.001
	agree	258	36.44		
	Neutral	148	20.90		
	Disagree	59	8.33		
	Strongly disagree	45	6.36		

**Table 5:** Primary concern regarding the consumption of foods obtained from animals fed insect-based protein (*n* = 708).

Questions	Reason *	Numbers (*n*)	Ratio %	Chi^2^ Value	*p*-Value
In case you are unwilling/do not wish to consume food products from animals fed on insect protein, the reason would most likely	No concerns	328	46.33	99.333	0.001
	Insufficient knowledge or need for additional information	121	17.09		
	Religious considerations	99	13.98		
	Feelings of disgust	81	11.44		
	Food safety concerns	66	9.32		
	Potential risk of allergic reactions	6	0.85		
	Concerns about alterations in the sensory attributes of the product	5	0.71		
	Other factors	2	0.28		
Total		708	100		

* Respondents selected one response option representing their primary concern. This was a structured, closed-ended item; no free-text responses were collected.

## Data Availability

The de-identified data supporting the quantitative findings of this study are available from the corresponding author upon reasonable request, subject to applicable ethical and institutional restrictions. The questionnaire did not collect open-ended qualitative responses.

## Supplementary Materials

The following supporting information can be downloaded at https://www.mdpi.com/article/10.3390/foods15213891/s1. The full questionnaire used in this study is provided in the Supplementary Materials in its original Arabic. It reproduces all sections, items, response options, and the standardized information text that participants received before answering the attitude and acceptance questions, exactly as administered in the online survey.
