# Supplementary material for: Assessing Consumer Perceptions of Foods from Insect-Fed Animals in the Kingdom of Saudi Arabia: A Cross-Sectional Study
Source: Foods. 2026 Oct 30;15(21):3891. doi: 10.3390/foods15213891 (PMC13763240; doi:10.3390/foods15213891)
Supplement: Supplementary file 1 [file foods-15-03891-s001.zip › foods-4573110-supplementary.pdf]

Qassim University جامعة القصيم

الهيئة العامة للغذاء والدواء
Saudi Food & Drug Authority

"استبيان قياس مدى تقبل المستهلكين لاستهلاك منتجات غذائية من حيوانات مُغذّاة على بروتين حشري في المملكة العربية السعودية"

الهدف من الاستبيان:

- قياس مدى تقبل المستهلكين لاستهلاك منتجات غذائية من حيوانات مُغذّاة على بروتين حشري في المملكة العربية السعودية.
- أنت مدعو للمشاركة في هذا الاستبيان والذي يستغرق اكماله ما بين 5-10 دقائق.
- المشاركة في هذا الاستبيان طوعية، ونؤكد بأن جميع البيانات محاطة بالسرية وستستخدم من قبل الباحثين لغرض النشر والبحث العلمي فقط.
- في حال الموافقة، نرجوا البدء بتعبئة الاستبيان أدناه:

1. البيانات الديموغرافية:

1 .1 الجنس: ذكر ☐ أنثى ☐

1.2 العمر: 18-29 ☐ 30-44 ☐

45-59 ☐ 60 فأكثر ☐

1.3 المستوى التعليمي: ثانوي أو أقل ☐ جامعي ☐

دراسات عليا ☐

1.4مكان الإقامة: المنطقة الوسطى ☐ المنطقة الشرقية ☐

المنطقة الغربية ☐ المنطقة الشمالية ☐

المنطقة الجنوبية ☐

1.5 الدخل الشهري: لا يوجد دخل ☐ (أقل من 5000 ريال)☐

(من 5000 ريال الى أقل من 10000 ريال)☐

أفضل عدم الإفصاح ☐ (10000 ريال فأكثر)☐

---

2. المعرفة المسبقة للمستهلكين: (يهدف التعرف على مستوى وعي المستجيب بالموضوع قبل أي تأثير خارجي)

2 .1 هل سمعت مسبقاً عن استخدام البروتين الحشري كمصدر للبروتين في تغذية الحيوانات؟

- نعم ☐
- لا ☐

2 .2 إذا كانت الإجابة نعم، فما مصدر معرفتك؟ يرجى وضع علامة (✓)

- وسائل التواصل الاجتماعي ☐
- أخبار أو مقالات ☐
- دراسات أكاديمية ☐
- من الأصدقاء أو العائلة ☐
- أخرى: ________

2 .3 هل تعلم أن تغذية الحيوانات على بروتين حشري تحقق فوائد غذائية (مثل المحتوى البروتيني المرتفع) وأيضاً بيئية؟

- نعم ☐
- لا ☐

2 .4 قبل معرفة أي معلومات، هل لديك تحفظات تجاه استهلاك منتجات حيوانية من حيوانات مُغذّاة على بروتين حشري؟

- نعم ☐
- لا ☐
- غير متأكد ☐

2 .5 هل تعلم أن البروتين الحشري يُعد جزءاً طبيعياً من النظام الغذائي لبعض الحيوانات (مثال: الديدان للطيور، أو اليرقات للأسماك، أو بعض أنواع الحشرات بالنسبة للدواجن)

- نعم ☐
- لا ☐

---

3. مرحلة تقديم فوائد استخدام البروتين الحشري للمستجيبين.

يُعد البروتين الحشري مصدر بروتيني في أعلاف الحيوانات من البدائل البروتينية الواعدة، نظرًا لارتفاع محتواه من البروتين واحتوائه على الأحماض الأمينية الأساسية. كما يحتوي على أحماض دهنية مفيدة، بما في ذلك حمض الأوميغا-3، مما يجعله خيارًا تغذويًا مناسبًا في صناعة الأعلاف عند إنتاجه ضمن ضوابط صحية وتنظيمية معتمدة.

وتشير الدراسات إلى إمكانية استخدامه كبديل لبعض مصادر البروتين التقليدية مثل فول الصويا أو مسحوق السمك في الأعلاف، مع الحفاظ على القيمة الغذائية لأعلاف الحيوانات.

وعلى الصعيد البيئي، يتطلب إنتاج البروتين الحشري مساحات أرضية ومياه وكميات علف أقل مقارنة ببعض المحاصيل البروتينية التقليدية، مما قد يسهم في تقليل الاحتباس الحراري والانبعاثات المرتبطة بالإنتاج الحيواني ويدعم مفاهيم الاستدامة والأمن الغذائي.

---

4. المواقف تجاه استهلاك منتجات حيوانية مغذاة على بروتين حشري. (الإجابة بعد توعية المستجيب بفوائد البروتين الحشري)

- ما مدى موافقتك على العبارات التالية باستخدام مقياس من 1 إلى 5، حيث 1 = لا أوافق بشدة، 5= أوافق بشدة:

4.1: معرفة أن استخدام البروتين الحشري كمصدر بروتيني بديل في الأعلاف آمن ولن يضر بصحتي تزيد من احتمالية استهلاكي لهذه المنتجات:

1- لا أوافق بشدّة ☐ 2-لا أوافق ☐ 3- محايد ☐ 4- أوافق ☐ 5- أوافق بشدّة ☐

4.2: أشعر بالراحة اذا عرفت بأن هذه المنتجات آمنة ومعتمدة من قبل الجهات الرسمية:

1- لا أوافق بشدّة ☐ 2-لا أوافق ☐ 3- محايد ☐ 4- أوافق ☐ 5- أوافق بشدّة ☐

4.3: الفوائد البيئية ستشجعني على استهلاك منتجات من حيوانات مُغذّاة على بروتين حشري:

1- لا أوافق بشدّة ☐ 2-لا أوافق ☐ 3- محايد ☐ 4- أوافق ☐ 5- أوافق بشدّة ☐

4.4: أرى أن استخدام البروتين الحشري كعلف يساهم في الاقتصاد الدائري ويعزز من الاستدامة والأمن الغذائي في المملكة العربية السعودية:

1- لا أوافق بشدّة ☐ 2-لا أوافق ☐ 3- محايد ☐ 4- أوافق ☐ 5- أوافق بشدّة ☐

4.5: سأشتري منتجات حيوانية من حيوانات مُغذّاة على بروتين حشري إذا كانت حلالًا وفق الضوابط الشرعية في المملكة العربية السعودية:

1- لا أوافق بشدّة ☐ 2-لا أوافق ☐ 3- محايد ☐ 4- أوافق ☐ 5- أوافق بشدّة ☐

4.6: أعتقد أن استبدال مصادر البروتين المعدّلة وراثيًا بالبروتين الحشري في تغذية الحيوانات يزيد من تقبلي لاستهلاك لحوم هذه الحيوانات:

1- لا أوافق بشدّة ☐ 2-لا أوافق ☐ 3- محايد ☐ 4- أوافق ☐ 5- أوافق بشدّة ☐

4.7: سأقبل استهلاك لحم أو بيض من حيوانات مُغذّاة على بروتين مستخلص من الحشرات:

1- لا أوافق بشدّة ☐ 2-لا أوافق ☐ 3- محايد ☐ 4- أوافق ☐ 5- أوافق بشدّة ☐

4.8: سأشتري منتجات حيوانية من حيوانات مُغذّاة على بروتين حشري إذا كانت متوفرة في السوق:

1- لا أوافق بشدّة ☐ 2-لا أوافق ☐ 3- محايد ☐ 4- أوافق ☐ 5- أوافق بشدّة ☐

4.9: أعتقد أنني سأشتري منتجات حيوانية من حيوانات مُغذّاة على بروتين حشري إذا كان سعرها أقل من المنتجات التقليدية:

1- لا أوافق بشدّة ☐ 2-لا أوافق ☐ 3- محايد ☐ 4- أوافق ☐ 5- أوافق بشدّة ☐

4.10: أوافق على دفع سعر أعلى قليلًا إذا كانت هذه المنتجات الحيوانية من حيوانات مُغذّاة على بروتين حشري مفيدة لصحة الإنسان :

1- لا أوافق بشدّة ☐ 2-لا أوافق ☐ 3- محايد ☐ 4- أوافق ☐ 5- أوافق بشدّة ☐

4.11: أوافق على دفع سعر أعلى قليلًا إذا كانت هذه المنتجات الحيوانية من حيوانات مُغذّاة على بروتين حشري تساهم في الحفاظ على البيئة وتقليل الانبعاثات:

1- لا أوافق بشدّة ☐ 2-لا أوافق ☐ 3- محايد ☐ 4- أوافق ☐ 5- أوافق بشدّة ☐

4.12: لن أشتري هذه المنتجات إذا كان السعر أعلى من سعر المنتجات التقليدية بنسبة أكثر من 10%:

1- لا أوافق بشدّة ☐ 2-لا أوافق ☐ 3- محايد ☐ 4- أوافق ☐ 5- أوافق بشدّة ☐

4.13: سأجرب هذه المنتجات إذا أُتيحت لي فرصة تجربة مجانية:

1- لا أوافق بشدّة ☐ 2-لا أوافق ☐ 3- محايد ☐ 4- أوافق ☐ 5- أوافق بشدّة ☐

4.14 : بشكل عام, أتقبل استهلاك منتجات حيوانية من حيوانات مُغذّاة على بروتين حشري:

1- لا أوافق بشدّة ☐ 2-لا أوافق ☐ 3- محايد ☐ 4- أوافق ☐ 5- أوافق بشدّة ☐

---

5. العقبات والمخاوف.

5.1 في حالة ممانعتك/عدم رغبتك تناول منتجات غذائية من حيوانات مُغذّاة على بروتين حشري، سيكون السبب غالباً عائد إلى: (يمكنك اختيار أكثر من إجابة)

-لا توجد لدي مخاوف ☐

-حاجتي إلى معلومات إضافية/ قلة المعرفة ☐

-أسباب دينية ☐

-الاشمئزاز ☐

-قلق حيال سلامة المنتجات الغذائية ☐

-أشعر بالقلق لاحتمالية الإصابة بردود فعل تحسسية ☐

-قلق مرتبط بتغيّر الخصائص الحسية للمنتج الغذائي (مثل: الطعم والرائحة والنكهة والقوام) ☐

5.2أسباب أخرى أو ملاحظات شخصية.

إذا كان لديك أسباب إضافية تمنعك من استهلاك منتجات حيوانية من حيوانات مغذاة على بروتين حشري، يرجى كتابتها هنا: ____

نقدّر لك مساهمتك في الإجابة على هذا الاستبيان، حيث أنها تساعد الباحثين على فهم مدى تقبّل المستهلكين لاستهلاك منتجات حيوانية مغذاة على بروتين حشري. نؤكد أن جميع إجاباتك ستظل سرية وسيتم استخدامها فقط لأغراض البحث العلمي.

شكراً لحسن تعاونكم.
